# Cross-reactive immunity potentially drives global oscillation and opposed alternation patterns of seasonal influenza A viruses

**DOI:** 10.1038/s41598-022-08233-w

**Published:** 2022-05-25

**Authors:** Lorenzo Gatti, Mischa H. Koenen, Jitao David Zhang, Maria Anisimova, Lilly M. Verhagen, Martin Schutten, Ab Osterhaus, Erhard van der Vries

**Affiliations:** 1 Institute of Applied Simulations, Zürich University of Applied Sciences, Einsiedlerstrasse 31a, 8820 Wädenswil, Switzerland; 2 Institute of Molecular Life Sciences, University of Zürich, Winterthurerstrasse 190, 8057 Zurich, Switzerland; 3 SIB Swiss Institute of Bioinformatics, Quartier Sorge-Batiment Genopode, 1015 Lausanne, Switzerland; 4 Department of Pediatric Infectious Diseases and Immunology, Wilhelmina Children’s Hospital, University Medical Center Utrecht, Lundlaan 6, 3584 EA Utrecht, The Netherlands; 5 Roche Pharma Research and Early Development, Pharmaceutical Sciences, Roche Innovation Center Basel, F. Hoffmann-La Roche Ltd, Grenzacherstrasse 124, 4070 Basel, Switzerland; 6 Department of Viroscience, Erasmus University Medical Center, Doctor Molewaterplein 40, 3015 GD Rotterdam, The Netherlands; 7 Research Center for Emerging Infections and Zoonoses, Veterinary University Hannover, Bünteweg 9, 30559 Hannover, Germany; 8 Artemis One Health, Molengraaffsingel 10, 2629 JD Delft, The Netherlands; 9 Department of Infectious Diseases and Immunology, Virology Division, Faculty of Veterinary Medicine, Utrecht University, Yalelaan 1, 3584 CL Utrecht, The Netherlands; 10 Clinical Chemistry and Hematology, University Medical Center Utrecht, Heidelberglaan 100, 3584 CX Utrecht, The Netherlands; 11 Research and Development, GD Animal Health, Arnsbergstraat 7, 7418 EZ Deventer, The Netherlands

**Keywords:** Immunology, Virology

## Abstract

Several human pathogens exhibit distinct patterns of seasonality and circulate as pairs. For instance, influenza A virus subtypes oscillate and peak during winter seasons of the world’s temperate climate zones. Alternation of dominant strains in successive influenza seasons makes epidemic forecasting a major challenge. From the start of the 2009 influenza pandemic we enrolled influenza A virus infected patients (*n* = 2980) in a global prospective clinical study. Complete hemagglutinin sequences were obtained from 1078 A/H1N1 and 1033 A/H3N2 viruses. We used phylodynamics to construct high resolution spatio-temporal phylogenetic hemagglutinin trees and estimated global influenza A effective reproductive numbers (*R*) over time (2009–2013). We demonstrate that *R* oscillates around *R* = 1 with a clear opposed alternation pattern between phases of the A/H1N1 and A/H3N2 subtypes. Moreover, we find a similar alternation pattern for the number of global viral spread between the sampled geographical locations. Both observations suggest a between-strain competition for susceptible hosts on a global level. Extrinsic factors that affect person-to-person transmission are a major driver of influenza seasonality. The data presented here indicate that cross-reactive host immunity is also a key intrinsic driver of influenza seasonality, which determines the influenza A virus strain at the onset of each epidemic season.

## Introduction

Several human respiratory viruses circulate as groups of discrete pathogenic entities exhibiting distinct patterns of seasonality^1,2^. For influenza virus such patterns have been studied extensively^3–5^. In the world’s temperate climate zones influenza activity oscillates and synchronizes with winter periods, while in tropical regions activity appears to be year-around or split into different seasons^4^. They have been attributed largely to ‘extrinsic’ factors driving efficient virus spread^6^, like air humidity variations^7^, seasonal influences on host susceptibility^8^, and societal structure and behavioural patterns^9^. However, several other mechanisms focussed on host and population immunity have been proposed that explain the seasonality of viruses. Firstly, during an epidemic or pandemic season the viral spread of a specific viral subtype will increase up to a point when herd immunity will prevent further transmission. The loss of specific immunity within a population can lead to a large peak of infections, as was seen for respiratory syncytial virus (RSV) in children after an almost absent RSV-season due to the restrictions of the COVID19-pandemic^10,11^. Apart from subtype specific immunity, Susceptible–Infection–Recovery epidemiological modelling predicted that also cross-reactive immunity plays a role in seasonality of viruses. These models postulate that the presence of a short-term cross-reactive host immune-phenomenon could explain a temporary reduction in host susceptibility to other viruses and cause seasonal infection patterns at a population level^12,13^. Thirdly, in recent years mathematical simulations that mimic virus dynamics suggest the existence of virus-virus interactions. These interactions show that the rise in incidence of infections caused by one virus directly influences the amount of other virus infections at both population as well as individual host level. This phenomenon was also found in clinical cohort where they found that the presence of rhinovirus interfered with the presence of Influenza A virus in patients^14,15^. This direct interaction between viruses caused one virus to block the other and thereby affect the seasonality of viruses.

All of these ‘intrinsic’ factors may also be attributed to other aspects of influenza epidemiology, like the replacement of a seasonal strain by a pandemic virus. This occurred for the last time during the 2009 influenza pandemic when the seasonal A/H1N1 was replaced by the pandemic A/H1N1 virus. Interestingly, like the other known pandemics this virus did not spread in winter, but during the 2009 northern hemisphere summer^16^.

**Figure 1:**
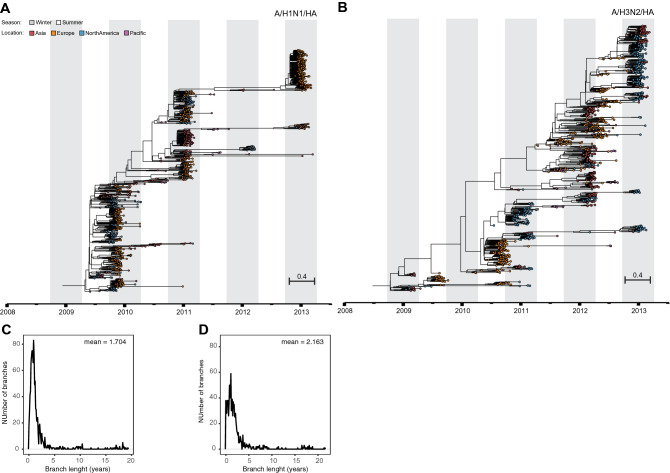
Spatio-temporal resolved phylogenies reveal intrinsic evolutionary influenza dynamics. Influenza hemagglutinin tree inferred by birth–death skyline phylodynamic modelling using 1078 (A/H1N1) (**A**) and 1033 (A/H3N2) (**B**) complete gene sequences. Distribution of average trunk-to-tips branch lengths of A/H1N1 (**C**) and A/H3N2 (**D**) phylogenetic trees.

To date, the newly introduced pandemic 2009 A/H1N1 virus continues to co-circulate with the A/H3N2 subtype causing seasonal epidemics in humans. Both influenza A viruses are under intense selective pressure by the host immune system and they continuously evolve to persist in humans. Viruses escape from pre-existing immunity through mutation at antigenic sites at the globular head of the hemagglutinin (HA). This is a major virus surface glycoprotein and primary target of host neutralizing antibodies. Continual viral presence in the population on the other hand results in a ‘landscape of immunity’^12^, which new ‘antigenic drift’ viruses need to overcome to fuel new epidemics. A typical phylogenetic tree of HA is shaped, as a result of this cat-and-mouse game, into a single trunk tree with short-lived branches^17^ (Fig. 1).

Virus strains that are antigenically similar cluster along the trunk of the tree with only a limited number of amino acid positions involved in the jump from an existing into a new antigenic cluster^18^. These positions were previously identified with data obtained from the hemagglutination inhibition assay, a serological test to assess neutralizing antibody responses to HA.

Besides these long-lived and predominantly strain-specific antibody-mediated immune responses, a shorter-lived, non-specific component has been proposed in particular to explain the limited virus genealogical diversity (single trunk) and lifespan (short-lived branches) of the vast majority of circulating dead-end virus lineages^12^. Evidence for such component has first come from in vitro and animal studies showing that pre-infection with one subtype induces partial cross-protection from infection with another subtype^19^. Observational studies addressing the potential role of cross-reactive immunity in global influenza seasonality has so far failed to show a clear pattern^20,21^. However, recent and marked observations to support a major role of cross-immunity were related to the fast disappearance of the A/H1N1 subtype from 1977, shortly after the introduction of the pandemic influenza A/H1N1 virus in 2009, while the A/H3N2 subtype managed to continue its circulation^13^. Another example was seen in the winter of 2018–2019 where in a matter of weeks A/H1N1 dominance was almost completely replaced by A/H3N2 dominance^22^.

## Results

In search for the existence of such component we first followed a phylodynamic approach to jointly resolve spatio-temporal phylogenetic HA trees of A/H1N1 and A/H3N2 subtypes and to infer underlying host population dynamics^23,24^ (Fig. 1; Tables S1 and S2). The dataset used here had been collected globally during the first 5 years after the onset of the 2009 influenza pandemic. It enrolled patients year-around (> 1 year of age), the vast majority (> 97%) with uncomplicated and PCR-confirmed influenza, who had been admitted—within 48 h after symptom onset—to primary care centres and hospitals in Asia (Hong Kong; *n* = 6), Europe (*n* = 37), the US (*n* = 36) and the Pacific (Australia; *n* = 8) (Fig. S1). From these samples 2111 influenza A viruses were isolated, which allowed us to obtain complete HA sequences from 1078 A/H1N1 and 1033 A/H3N2 viruses. The extent of sampling, directly after the pandemic outbreak, in combination with an unprecedented resolution regarding quality-controlled Sanger sequencing, resulted in a high-resolution dataset. This offered us an unique window of opportunity to study the dynamics of the estimated effective reproductive number *R* over time (*R*-skylines). *R* is a parameter of host immunity^25^, and is computed here as the rate at which an infected individual gives rise to a new infection in a defined period of time.

We observed that *R-*skylines estimated from the A/H1N1 and A/H3N2 trees showed alternate phases of increasing and declining *R*, with *R* < 1 and *R* > 1 respectively (Fig. 3). There was a significant negative correlation between phases (Pearson’s ρ = − 0.511, *P* = 3.0e−07; D = 0.202, *P* = 0.052) with an average endogenous oscillation period estimated to be approximately 1.67 ± 0.01 years for A/H1N1 and 1.13 ± 0.02 years for A/H3N2^6^ as computer by periodograms (Fig. S4). Of note, these periods were similar to the average lifespan of the dead-end virus lineages on the HA trees (1.7 ± 0.4 for A/H1N1 and 2.2 ± 0.5 for A/H3N2) (Fig. 1).

Given the finite nature of susceptible hosts, virus persistence relies on the availability of new susceptible ones, which forces viruses to migrate between geographical locations^26^. The interplay between antigenic drift and pre-existing immunity may then determine the outcome of the competition between these viruses at the onset of each influenza season^27^. As the observed pattern of *R*-skylines indicates that a relatively short-lived cross-reactive immunity component drives subtype competition at the host scale, we wondered whether this competition also could determine the dynamics of global viral spread.

To build on existing global viral spread data and to study its patterns for the A/H1N1 virus after 2009 we expanded our dataset with complete HA sequences deposited in the IRD from viruses isolated prior to (2008–2009) and after (2013–2015) our study period (Dataset S1). We then inferred the number of geographical location changes at each internal node of these trees to identify all virus movements from one geographical location (source) to another location (sink). Again, and similar to the *R-*skylines, influenza A/H1N1 and A/H3N2 viral spread alternated globally within our study period (Fig. 4). Global influenza A/H1N1 viral spread dominated in the first half of the study period, while A/H3N2 viral spread were more prevalent between 2012 and 2013. The observation of oscillation between influenza A/H1N1 and influenza A/H3N2 throughout the study period supports the evidence that inter-subtype competition presented here exists on both the population and individual host level, contributes to influenza seasonality and may determine the virus subtype that will dominate in a given influenza season.

**Figure 2:**
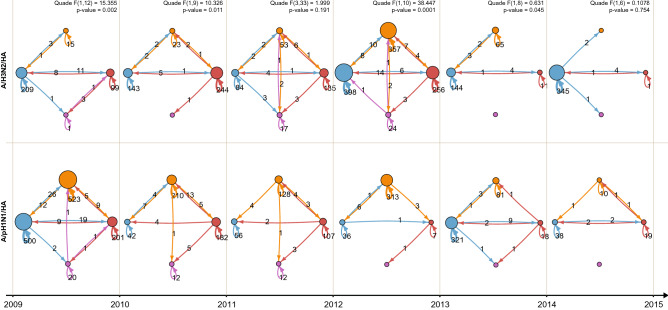
Network reconstruction of viral spread between the sampled geographic locations show increased spread of influenza A/H1N1 (bottom row) compared with influenza A/H3N2 (top row) between 2009–2011, followed by an opposite trajectory between 2011–2013. Inferred viral spread networks between geographic locations are depicted between centers located in Asia (red), Europe (orange), North America (blue) and Pacific (purple). Viral spread events during a 1-year time window were pooled and numbers were shown on the arrows. The diameter of the nodes is proportional to the number of sink viral spread events, while the arrow width is proportional to the number of source spread events. The Quade test and correspondent post-hoc procedures were applied to test significant differences between spread trends and preferred viral spread trajectories are presented at the top. Significance level was set to 0.05.

Finally, previous work on global circulation had shown that East and South-East Asia (E-SEA) played a pivotal role in global dissemination of A/H3N2 viruses. Here, A/H3N2 virus activity was found year-round (between 2000 and 2012), from where new antigenic drift variants fuelled in the temperate climate zone epidemics^9^. In contrast, E-SEA did not seem to have a major role in the dissemination of pre-pandemic A/H1N1 viruses^9^. To study global virus spread after 2009 we constructed the networks of viral spread trajectories between the sampled geographical locations using measures of connectivity (Fig. S2) in a one-year time window (Figs. 2 and 4) and found that, in contrast to the pre-pandemic period^9^, E-SEA was equally important for the dissemination of both influenza A viruses. Within our dataset we examined the distribution of migration events between different locations and counted the amount of location switches on the tree. We counted 56 A/H1N1 and 58 A/H3N2 dissemination events from E-SEA to the other sampled regions in the world (Fig. S3). In addition, global viral spread patterns showed a similar degree of global network complexity (Fig. S2, max. graph density/diameter of 1.08/12.09 for A/H1N1 and 1.16/10.64 for A/H3N2) and similar patterns of virus circulation across the sampled geographic regions (Fig. S2, max. number of islands and graph reciprocity of 2 and 0.75 for A/H1N1 and 2 and 0.86 for A/H3N2).

## Discussion

**Figure 3:**
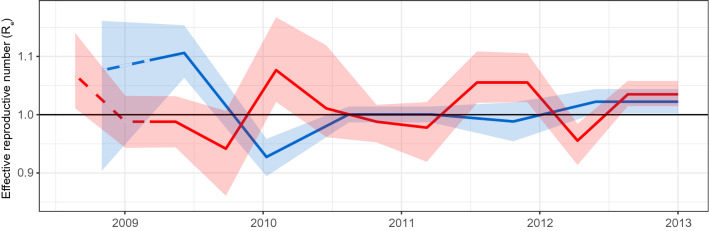
Oscillation of reproductive number *R*-skylines estimated from influenza A virus phylogenies with opposed alternation of phases between subtypes. Time-series (2009–2013) for influenza A/H1N1 (blue, *n* = 1078) and A/H3N2 (red, *n* = 1033) viruses. Pre-pandemic period is indicated with dashed lines. Shaded regions represent 95% Highest Posterior Density interval.

Extrinsic factors probably play a role in forcing influenza epidemics into the winter seasons in the global temperate climate zones^3–5,7^. The oscillating and alternating pattern of the global skylines of *R* we present here are consistent with the notion that cross-reactive host immunity is an important intrinsic driver of influenza seasonal patterns (Fig. 3).

**Figure 4:**
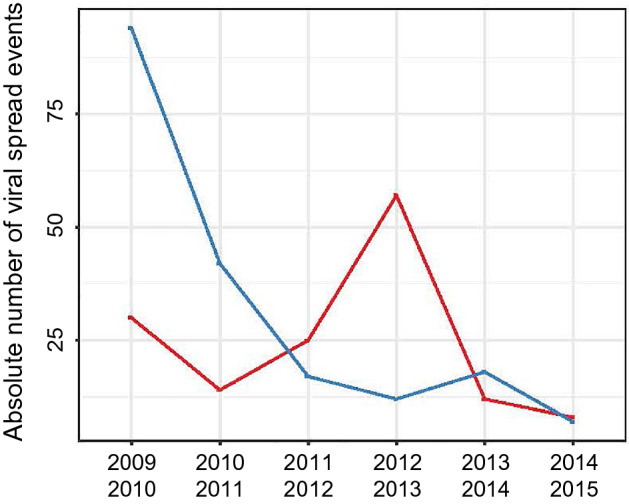
Opposed alternation of influenza A virus viral spread events. Total number of viral spread events between different geographical locations of influenza A/H1N1 (blue, *n* = 190) and A/H3N2 (red, *n* = 146) viruses were pooled by 1-year intervals from the start of the 2009 pandemic. Viral spread counts were performed by traversing the fully-spatiotemporal-resolved phylogenetic trees in post-order.

Global influenza dissemination dynamics also reveals alternation of global virus spread and complexity of viral spread trajectories between subtypes with two phases (Figs. 2 and 4). In the first phase (2009–2011) we observe that these parameters are high for the A/H1N1 and low for the A/H3N2 subtype (Figs. 2 and 4). This pattern is reversed during the second phase (2011–2013). These parameters are indicators of virus persistence and depend, therefore, on the availability of susceptible hosts within a defined geographical location^26^. This implies that an intrinsic correlation exists between change of cross-reactive host immunity landscapes and global viral spread. In the Netherlands patterns and duration for influenza epidemic periods similar to our results were previously found by were previously found. Additionally, statistical regression techniques showed that depletion of susceptible hosts was the most important factor in determining transmission of influenza virus during these epidemic^28^ as well as in seasonal corona outbreaks^29^. Seasonal patterns are also described between influenza virus and other respiratory viruses. In mathematical models that studied the co-circulation of seasonal influenza A virus and non-influenza respiratory viruses, a short-term protection of one virus on the other induced seasonal patterns^15,21^. Furthermore, in a clinical study, the presence of rhinovirus protected patients against infection with influenza A virus^14^. Taking into account the strong cross-reactive immune responses previously observed between the seasonal and pandemic A/H1N1 virus subtypes, cross-reactive immunity may well have forced the 2009 pandemic into the northern hemisphere summer and mitigated the disease burden associated with this virus^16,30,31^.

A great deal of the current understandings about influenza antigenic drift is based on data generated by the hemagglutination inhibition serological test. This assay is largely dependent on serum immunoglobulin G (IgG) content^32^, but it does not account for other immunity components of the immune system. The observed short endogenous oscillation periods observed (1.7 years for A/H1N and 1.1 years for A/H3N2) suggest that this is most likely the result of short-lived inter-subtypic immune responses rather than systemic antibody-mediated immunity from which major antigenic drift variants arise every few years (Fig. 3 and Fig. S4)^9,12,18^. T-cells have been suggested as key players in mediating inter-subtypic immunity through several mechanisms, including direct cytolytic activity and interactions with B cells^30,31^. Inter-subtypic short-lasting immune responses likely also exist for other viruses. Seasonal coronaviruses induce a short-lasting immunity of 12 months in individuals^29,33^. Furthermore, SARS-CoV-2 reactive CD4+ T cells were found in healthy SARS-CoV-2 seronegative individuals, most likely due to cross-reactive T cells that stemmed from previous infections with endemic seasonal coronaviruses^34^.

Finally, secretory immunoglobulin A (IgA), the most abundant antibody isotype, is an interesting candidate meeting all criteria: IgA mediated-immunity is relatively short-lived^35^, is (partially) cross-reactive^36^, and thought to be the main driver of upper respiratory tract mucosal immunity against influenza^37^. Moreover, intramuscular administration of IgA, and not IgG, prevented airborne virus transmission in the ferret and guinea pig model^32^, indicating that cross-reactive IgA levels may directly impact person-to-person spread of the virus^38^. Cross-reactive immunity to different influenza subtypes can be the result of antibodies that are able to bind to the highly conserved stalk of the HA protein. These antibodies get produced most upon sequential exposition to diverse HA subtypes^39^ and in this, IgA has a more potent neutralizing effect against influenza than IgG^40^. Further studies elucidating the contribution of host immunity to seasonality of influenza and other multi-strain viruses, such as the coronavirus, paramyxoviruses, respiratory syncytial virus and human metapneumovirus are warranted^41^. This would further support the establishment and exploitation of global virus and bio-banks^42^, which will lead to a better understanding of the contribution of host immunity landscapes to the dynamic epidemiological circulation patterns of (multi-strain) pathogens.

## Methods and materials

### Study conduct

IRIS (NCT00884117) is a prospective, multicentre, global observational study offering unprecedented resolution with regard to quality-controlled Sanger sequencing^43,44^. This report summarizes the results from 87 centers in, Australia (*n* = 8), China (Hong Kong, *n* = 6), Europe (*n* = 37; France, Germany, Norway) and the United States (*n* = 36) from December 2008 to March 2013, comprising five Northern and four Southern Hemisphere seasons, and including the 2009–2010 pandemic. Centers were selected to achieve the widest geographic coverage possible within each country (Fig. S1).

### Patient selection

Adults and children aged ≥ 1 year were included year-round (*n* = 2980; excluding 21 patients (1%) with mixed influenza A and B virus infections) in the study if they were influenza-positive by rapid test (QuickVue Influenza A + B Test; Quidel Corp) at presentation and/or had predefined clinical signs and symptoms of influenza for ≤ 48 h for hospitalized patients (no time limit for hospitalized children). The vast majority (> 97%) had uncomplicated influenza.

### Ethics approval

The study was performed in compliance with the principles of the Declaration of Helsinki and its amendments, and in accordance with Good Clinical Practice. Independent ethics committees and institutional review boards at each centre approved the study protocol and amendments. All patients or legal guardians provided written informed consent at the time of enrollment.

### Virus identification

Throat and posterior nasal swab specimens were obtained on day 1, 3, 6 and 10 and shipped on dry ice to a central laboratory for analysis (Erasmus MC, Rotterdam, The Netherlands). Influenza A subtypes were identified using semi-quantitative real-time reverse transcription polymerase chain reaction (RT-PCR)^45^. Day 1 samples with cycle threshold (Ct) values of < 32 were cultured on Madin–Darby canine kidney cells. Virus-containing supernatants were cleared from cell debris by centrifugation (10 min at 1000×g) and stored at − 80 °C until further processing. For this study, A/H1N1 (*n* = 1078) and A/H3N2 (*n* = 1033) virus isolates were included, which were obtained upon patient admission (day 1).

### Datasets and nucleotide sequence accession numbers

Sanger sequencing of hemagglutinin (HA) genes was done for all isolated viruses. Complete HA sequences were obtained from influenza A/H3N2 (*n* = 1033) and A/H1N1 (*n* = 1078) subtypes. These sequences and metafile data are provided in the Supplementary Information. To build on existing data on global influenza viral spread we expanded the IRIS dataset with all available complete HA and Neuraminidase sequences from the NIAID Influenza Research Database (IRD) collected between 2008–2009 and 2013–2015 in countries included in the IRIS study^46^. Numbers of additional HA sequences were 443 for A/H1N1 and 462 for A/H3N2 respectively. The complete list of IRD sequences is provided (Dataset S1).

### Data pre-processing and alignments

Each expanded dataset was aligned using ProGraphMSA using default parameters^47^. Sequences were renamed to include sampled geographical locations, sampling dates (continuous values) and corresponding influenza season (when available).

### Phylodynamics inference

The birth death serial skyline (BDSKY) phylodynamics model implemented in BEAST v.2.3.1 was applied to the IRIS and expanded datasets to infer spatio-temporal resolved phylogenies and epidemiological parameters^23,26,48^. Phylogenetic trees were estimated under the general-time-reversible model (GTR+) with Γ-distribution to model among-site rate variation^49^ (Fig. 1). A molecular clock rate prior was set to follow an uncorrelated log-normal distribution^50^. Internal node calibration was performed using tip sampling dates^51^. The BDSKY-model was set with the following parameter: the sampling rate prior for the influenza infected population/the real sampled population followed a *Beta *(*1, 999*) distribution; the prior probability of sampling an individual upon becoming non-infectious followed a *LogNorm *(*4.5*, *1.0*) distribution. In addition, tree dating was performed using tip dates while an uncorrelated log-normal clock rate prior was applied to handle uncertainties in the sample collection dates. Finally, the analysis was run long enough to obtain a sufficient effective sample size ESS > 200 for all parameters. The converged parameters of the BDSKY-model are listed in Supporting Information Tables S1 and S2. To assess global model robustness, we performed two independent runs of each analysis (for a total of 20 runs). Markov chain Monte Carlo (MCMC) parameter convergences were diagnosed with Tracer 1.6. Thinning of BEAST2 output files (tree files and parameter files) was done using in-house bash scripts. After accurate MCMC trace monitoring, the first 10% of MCMC steps were discarded as burn-in resulting in around 6000 trees per each dataset. TreeAnnotator v2.3.1 was used to produce Maximum Clade Credibility (MCC) trees^23^.

### Statistical analyses effective reproductive number

We estimated the effective reproductive number *R* using phylodynamics modelling as described above. The estimates of *R* allowed us to study the dynamics of virus spread within the population^52^. Values *R* < 1 indicate a decline of infections, while *R* > 1 indicates that the infection has increased its spreading in a more susceptible population. The skyline of *R* is used here to picture the underlying dynamics ‘shaping’ a phylogenetic tree^23,24,26^. The univariate distributions of *R* values, estimated with independent sampling frequency from each dataset, were grouped and smoothed via interpolation to compensate for intermediate missing values. First, Wald–Wolfowiz, and Bartel Rank non-randomness tests were applied on each *R* median time-series as well as its permuted version^53^. The same test was then applied on the pairwise intersection of *R* median time-series, and the statistical support was evaluated by re-computing the test on permuted *R* median time-series. Secondly, the pairwise maximum difference between *R* median time-series was computed applying the Kolmogorov–Smirnov test (KS-test). The two-sample KS-test was used to compare the cumulative distributions of two data sets^54^. The KS-test reports the maximum difference between two cumulative distributions (D) and it returns a *P* computing the KS statistics from all the possible permutation of the original data. The significance level was set at 0.001, so that two distinct *R* median time-series were considered to be drawn from different distributions when D ≥ 0.45. Next, the pairwise-correlation between *R* median estimates was evaluated by the Pearson's product moment correlation coefficient ($\rho$). Pearson’s product moment correlation coefficient ($\rho$) was tested using the Fisher’s Z transform with 95% confidence interval and significance level set at 0.005^55^. Exploratory analyses on the *R* median time-series were applied to qualitatively identify oscillation periods and amplitude. The oscillation period of each *R* median time-series was then computed from the highest frequency value shown by the smoothed periodogram using the IRIS dataset. Statistical uncertainty on the inferred period was assessed from cumulative periodograms computed on 100 permutations of the original *R* median time-series. Finally, the overlap of HPD intervals of the pairwise *R* was computed for each R median time-series. The obtained value was then compared with the overlap of the HPD interval of *R* obtained with 100 permutations of the true HPD intervals.

### Viral spread routes and evolutionary rates

Datasets were partitioned according to the geographical sampling locations pooled by continent (North America, Europe, Asia, Pacific area). Viral spread rates were estimated using a discrete phylogeographic trait model with the Γ-distribution as substitution rate prior between geographical demes^56^. The influenza virus dissemination process was fitted to a discrete trait model using the Bayesian Stochastic Search Variable Selection method, by inferring the most parsimonious description of the phylogeographic diffusion process^57–60^. Counts of viral spread events were quantified by traversing the fully spatio-temporal resolved phylogenetic trees in post-order and by counting the number of most probable Markov chain jumps along the branches of the posterior set of trees^61,62^.

### Branch geographical persistence

Geographical persistence was quantified by summing the phylogenetic branch lengths (measured in expected substitutions per site) grouped by their inferred geographical location on the phylogenetic tree trunk (inferred traversing the phylogenetic tree from ‘leaf-to-root’ and summing the number of branch traversals. The tree trunk was defined as the path on the phylogenetic tree that has been traversed more than 10 times. Number of seeding events was defined as the number of switches on the phylogenetic tree trunk per season.

### Viral spread graphs

Trajectory networks were reconstructed per each strain variant, pooling viral spread events occurred within a one-year time window (Fig. 2). Trajectory complexity was computed estimating graph density, number of islands (nodes), diameter, and reciprocity^63,64^. In addition, geographical location connections were estimated by computing the graph centrality measures (specifically: degree centrality and betweenness centrality) (Figs. S2 and S3)^65–67^. The Quade and correspondent *post-hoc* procedures were applied to test whether viral spread trends were significantly different between strains and whether preferred viral spread trajectories were selected^68^. The significance level was set at 5%.

## Supplementary Information

Dataset S1.

Supplementary Information 1.

Supplementary Information 2.

Supplementary Information 3.

## Data Availability

Full-length HA sequences obtained as part of the IRIS study have been deposited in Genbank with the primary accession codes mentioned above. All other HA sequences downloaded for this study are listed in the Supportive information.

## Code availability

All source codes and BEAST .xml files are available on GitHub (http://www.github.com/gattil/IRIS-Influenza-Dynamics).
