# Supplementary material for: Cross-reactive immunity potentially drives global oscillation and opposed alternation patterns of seasonal influenza A viruses
Source: Sci Rep. 2022 May 25;12:8883. doi: 10.1038/s41598-022-08233-w (PMC9131982; doi:10.1038/s41598-022-08233-w)
Supplement: Supplementary file 1 — Dataset S1. [file 41598_2022_8233_MOESM1_ESM.pdf]

Dataset 1. Hemagglutinin genbank accession numbers used to expand the IRIS dataset

A/H1N1

AB514226,AB514227,AB558344,AB558381,AB558384,CY036669,CY036670,CY036690,CY039127,CY040114,CY040162,CY040637,CY041750,CY043086,CY043187,CY043792,CY044204,CY044909,CY044965,CY045013,CY045495,CY046331,CY046499,CY047326,CY049092,CY049188,CY049451,CY049883,CY049971,CY050174,CY050368,CY050420,CY050975,CY051087,CY051215,CY051423,CY051439,CY051463,CY051759,CY052015,CY052106,CY052154,CY052927,CY053158,CY053293,CY054548,CY054707,CY054827,CY054987,CY055019,CY055214,CY055764,CY055836,CY055876,CY055916,CY056036,CY056164,CY056619,CY056651,CY056723,CY056907,CY057027,CY057294,CY057430,CY057494,CY057678,CY057766,CY057966,CY058054,CY058548,CY060726,CY061946,CY062218,CY062402,CY062415,CY062421,CY062522,CY062643,CY062843,CY062875,CY063027,CY063163,CY063171,CY063235,CY063502,CY063566,CY063763,CY063795,CY064399,CY064440,CY064446,CY064628,CY065099,CY065920,CY067023,CY068994,CY069114,CY069266,CY069290,CY069397,CY071039,CY071103,CY071602,CY071642,CY072470,CY072558,CY072758,CY073378,CY073665,CY074131,CY074403,CY074427,CY074483,CY074571,CY075909,CY080618,CY080937,CY084454,CY087024,CY089187,CY092101,CY092928,CY099330,CY111659,CY111699,CY120445,CY120923,CY122695,CY122872,CY123174,CY123253,CY123268,CY123279,CY123561,CY123667,CY123736,CY123817,CY123929,CY123934,CY124079,CY128283,CY128347,CY147787,CY147939,CY147955,CY163463,CY163472,CY163474,CY163475,CY163478,CY163484,CY163491,CY163496,CY163500,CY163502,CY187193,CY187197,CY187204,CY187205,CY187221,CY187224,CY187227,CY187230,CY187234,CY187236,CY187240,CY187247,CY187248,CY187258,CY187261,CY187262,CY187269,CY187280,CY187293,CY187295,CY187296,CY187309,CY187311,CY187314,CY187328,CY187331,CY187338,CY187341,CY187350,CY187352,CY187360,CY187377,CY187636,CY187652,CY187655,CY187659,CY187670,CY187683,CY187707,CY187717,CY187725,CY188289,CY188313,CY188393,CY188537,CY188569,CY188889,CY188969,CY189033,CY189073,CY189169,CY189177,CY189209,CY189217,CY189297,CY189449,CY189529,CY189559,CY189735,EU516300,EU566976,EU779617,FJ686969,FJ984401,FJ998209,GQ162190,GQ162194,GQ168861,GQ221791,GQ225365,GQ232049,GQ323574,GQ329082,GQ338358,GQ338361,GQ359765,GQ377061,GQ377085,GQ466386,GQ475927,GQ527165,GQ894905,GQ896375,HM014332,HM144117,HM214478,HM230693,HM567816,HM780470,HQ228029,HQ228040,HQ228080,HQ228128,HQ291889,JN171945,JN256831,JN256842,JN256844,JN389446,JN412821,JN631054,JQ065113,JQ065149,JQ065150,JQ065169,JQ065208,JQ431208,JQ714172,JQ714175,JQ714177,KC780182,KC780246,KC780480,KC780489,KC780516,KC780981,KC781018,KC781042,KC781096,KC781103,KC781519,KC781905,KC781917,KC782046,KC782215,KF647985,KF648020,KF648032,KF648129,KF667891,KF667911,KF667919,KJ196070,KJ395995,KJ395999,KJ396002,KJ396010,KJ396012,KJ396014,KJ396024,KJ396028,KJ396031,KJ396032,KJ396036,KJ406387,KJ538498,KJ549778,KJ635894,KJ635902,KJ645781,KJ645784,KJ889280,KM186099,KM213259,KM219135,KM219159,KM219191,KM219279,KM366375,KM366455,KM366679,KM366759,KM408913,KM408982,KM409000,KM409009,KM409048,KM409069,KM409074,KM409077,KM409095,KM409101,KM409128,KM409134,KM409163,KM409190,KM409193,KM409226,KM409229,KM409241,KM409244,KM409262,KM409268,KM409271,KM409292,KM409319,KM409331,KM409367,KM409370,KM409385,KM437634,KM437642,KM437650,KM437834,KP019892,KP019901,KP019903,KP019911,KP019917,KP019923,KP019930,KP864371,KP864374,KP864375,KP864384,KP864394,KP864406,KP864411,KP864412,KP941705,KP941707,KP941712,KP941719,KP941720,KP941721,KP941724,KP941726,KP941727,KP941733,KP941737,KR271551,KR271583,KR271591,KR271599,KR611204,KR611211,KR611225,KR611229,KR611234,KR611235,KR611238,KT181075,KT181095,KT181100,KT181121,KT181135,KT181176,KT274236,KT274237,KT274240,KT274243,KT274282,KT274288,KT274296,KT274302,KT274312,KT274322,KT274326,KT274355,KT274363,KT274364,KT274374,KT274393,KT274409,KT274425,KT274449,KT274469,KT274474,KT274486,KT274493,KT274505,KT274513,KT274520,KT274522,KT274534,KT274553,KT274572,KT274583,KT274588,KT274606,KT274607,KT274608,KT274631,KT274640,KT274661,KT836462,KT836729,KT836731,KT836732,KT880114,KT880115,KT880131,KU051431,LC032825,LC032833,LC033097,LC033273,LC033281

A/H3N2

CY035054,CY036927,CY036951,CY036967,CY037311,CY037319,CY037519,CY037543,CY037575,CY

037591,CY037615,CY037631,CY037831,CY037863,CY037871,CY038815,CY038823,CY038847,CY038863,CY039431,CY041466,CY044429,CY044612,CY044620,CY044668,CY044748,CY044780,CY044812,CY044844,CY044852,CY050093,CY050108,CY050110,CY050127,CY050136,CY050452,CY050492,CY050540,CY050580,CY050620,CY050628,CY050692,CY050716,CY050732,CY050788,CY050796,CY050804,CY050812,CY058788,CY058804,CY061898,CY064855,CY067213,CY067937,CY067945,CY067969,CY067985,CY067993,CY068033,CY068041,CY068057,CY068145,CY068169,CY068209,CY068241,CY068265,CY068329,CY068361,CY068393,CY068457,CY068473,CY068481,CY068497,CY068521,CY068537,CY068545,CY068593,CY068617,CY068678,CY068718,CY068742,CY068750,CY068774,CY068782,CY068814,CY068822,CY068838,CY068854,CY069357,CY069429,CY080563,CY081454,CY084334,CY087159,CY089629,CY089773,CY091853,CY091855,CY091857,CY092329,CY092377,CY093303,CY093327,CY093343,CY093351,CY100073,CY100087,CY104622,CY105830,CY105862,CY106608,CY106632,CY106648,CY106712,CY106728,CY106736,CY106744,CY106752,CY106760,CY106792,CY106872,CY106896,CY106904,CY106992,CY113021,CY115528,CY115560,CY115584,CY115592,CY115608,CY115616,CY115624,CY115688,CY115720,CY115776,CY115784,CY119034,CY147667,CY172175,CY173191,CY173223,CY173263,CY173511,CY173519,CY173527,CY173559,CY187382,CY187645,CY187953,CY188089,CY188225,CY188249,CY188497,CY188545,CY189639,CY189655,CY189695,CY189823,CY193262,CY193264,CY193266,CY193270,CY193275,CY193281,CY193282,CY193297,CY193300,CY193307,CY193311,CY193317,CY193319,CY193320,CY193326,CY193329,CY193330,CY193363,CY193365,CY193374,CY193390,CY193408,CY193418,CY193422,CY193466,CY193469,CY193475,CY193478,CY193488,CY193496,CY193512,CY193519,CY193521,CY193529,CY193530,CY193541,CY193551,CY193556,CY193559,CY193565,CY193576,CY193580,CY193596,CY193613,CY193614,CY193619,CY193621,CY193660,CY193661,CY193690,CY193731,CY193747,CY193879,CY193950,CY193951,CY193976,CY193981,CY193982,EU716453,EU716462,EU716470,EU716471,EU779510,EU779516,EU779518,EU779520,EU779526,EU779532,EU851995,EU852001,EU852005,EU885499,EU885503,EU885512,EU885518,EU885524,EU885526,EU885528,EU885530,EU885532,EU885536,EU885538,EU914859,FJ009471,FJ009473,FJ179354,FJ179356,FJ532080,FJ686935,FJ686937,FJ686946,GQ293083,GQ369906,GQ369928,GQ385818,GQ385869,GQ385872,GQ385874,GQ385887,GQ385889,GQ385897,GQ385900,GQ385915,GQ895010,GQ895019,GQ895050,GQ902793,GQ902817,GQ902825,JN256696,JN256698,JN256700,JN256707,JN256709,JN256712,JN256722,JN256724,JN256725,JN256729,JN256741,JX437710,JX437829,KC535327,KC535342,KC535352,KC535369,KC535378,KC535381,KC535402,KC535417,KF014160,KF014197,KF014202,KF014230,KF014232,KF789774,KF789858,KF790136,KF790155,KF790297,KJ196077,KJ439217,KJ561706,KJ609206,KJ667973,KJ734749,KJ938665,KJ938671,KM061041,KM063935,KM063949,KM063954,KM063985,KM063992,KM064041,KM064092,KM064105,KM064114,KM064125,KM064140,KM064169,KM064187,KM064200,KM064201,KM064239,KM064254,KM064261,KM064272,KM064290,KM064315,KM064354,KM064362,KM064373,KM064376,KM064390,KM064413,KM064470,KM064501,KM064521,KM069488,KM069492,KM069494,KM069495,KM069497,KM069585,KM069595,KM069596,KM069600,KM069601,KM507567,KM852887,KM852927,KM852974,KM852976,KM853006,KM853009,KP701625,KP701631,KP701634,KP701636,KP701651,KP701657,KP701661,KP701669,KP701721,KP701727,KP877349,KP877360,KR057515,KR057558,KR057592,KR057637,KR057664,KR534277,KR534278,KT205932,KT277829,KT819393,KT836940,KT836943,KT836957,KT836992,KT837037,KT837039,KT837060,KT837104,KT837148,KT837180,KT837205,KT837286,KT837434,KT837526,KT837659,KT837668,KT837713,KT837715,KT837773,KT837779,KT837826,KT837837,KT837997,KT838021,KT838041,KT838053,KT838088,KT838131,KT838136,KT838329,KT838362,KT838396,KT838441,KT838545,KT838590,KT838599,KT838753,KT838765,KT838766,KT838833,KT838916,KT838921,KT838931,KT838939,KT838997,KT839006,KT839040,KT839200,KT839229,KT839231,KT839275,KT839370,KT839447,KT839524,KT839565,KT839585,KT839602,KT839615,KT839666,KT839714,KT839725,KT839893,KT839932,KT839994,KT840006,KT840176,KT840180,KT840226,KT840260,KT840344,KT840352,KT840421,KT840475,KT840525,KT840669,KT840689,KT840695,KT840718,KT840791,KT840926,KT840964,KT840984,KT840998,KT841007,LC033145,LC033329
