# Supplementary material for: Cross-reactive immunity potentially drives global oscillation and opposed alternation patterns of seasonal influenza A viruses
Source: Sci Rep. 2022 May 25;12:8883. doi: 10.1038/s41598-022-08233-w (PMC9131982; doi:10.1038/s41598-022-08233-w)
Supplement: Supplementary file 2 — Supplementary Information 1. [file 41598_2022_8233_MOESM2_ESM.docx]

| **List of Ethics Committees/Institutional Review Boards** |
| --- |
| **AUSTRALIA** |
| Sydney Children's Hospital Network, HRERC, Corner of Hawkesury Road, Westmead, NSW 2145 |
| WCHR HREC, Level 2, Samuel Way Building, 17 King William Road, North Adelaide, SA 50006 |
| Bellberry HREC, 229 Greehill Road, Dulwich, SA 5065 |
| South Eastern Sydney Research Network, Room G71, East Wing, Edmund Black Building, Prince of Wales Hospital, Randwick, NSW 2031 |
| Office for Research, Melbourn Health, PO Royal Melbourn Hospital, Parville, Victoria 3050 |
| Research Directorate, Monash Medical Centre, 246 Clayton Road, Clayton, Victoria 3168 |
| Queensland Health, Research Governance, Sunshine Coast Clinical School, Level 4, Block 3, Nambour General Hospital, Nambour, Queensland 4560 |
| Bellberry HREC, 229 Greehill Road, Dulwich, SA 5065 |
| **FRANCE** |
| Committee of Protection of People South-Est IV, Centre Leon Berard, 28 rue Laennec, 69373 Lyon Cedex 08 |
| **GERMANY** |
| Saxony Regional Medical Chamber, Schutzenhohe 16, PF 10 04 65, Dresden 01074 |
| Thuringian State Medical Board, P O Box 100740, Jena 07707 |
| Regional Medical Chamber Lower Saxony, Berliner Allee 20, Hannover 30175 |
| Regional Medical Chamber Baden-Wuerttemberg, Jahnstrasse 40, Stuttgart 70597 |
| **HONG KONG** |
| Joint Chinese University of Hong Kong New Territories East Cluster Clinical REC, 8/F, Lui Che Woo Clinical Science Building, POWH, Shatin |
| Institutional Review Board of the University of Hong Kong Hospital Authority, Hong Kong West Cluster, R 901, Administration Building, QMH |
| **THE NETHERLANDS** |
| Medisch Ethische Toetsings Commissie Erasmus MC, Postbus 2040, 3000 CA Rotterdam, Ta.v. het secretarieaat, Kamer Z514 |
| **NORWAY** |
| Regulatory Committee for Medical and Health Related Ethics, Postboks 7804, Bergen 5020 |
| **POLAND** |
| Komisja Bioetyczna przy Lubelskiej Izbie Lekarskiej, Chmielna 4 str., Lublin 20-097 |
| Bioethics Committee, 3 Czerwona Str, Lodz 93-005 |

| Komisja Bioetyczna przy Okręgowej Izbie Lekarskiej w Warszawie, Pulawska 18 str., W-wa 02-512 |
| --- |
| Komisja Bioetyczna przy ”ląskiej Izbie Lekarskiej w Katowicach, Grazynskiego 49a str., 40-126 Katowice |
| Komisja Bioetyczna przy Okręgowej Izbie Lekarskiej w Łodzi, Czerwona 3 Str. 93-005 Łódź |
| Bioethical Committee, 18 Pulawska Str, 02-515 Warsaw |
| Bioethical Committee, Regional Chamber of Physicians, Cracow Krupnicza Str. |
| Komisja Bioetyczna przy Centralnym Szpitalu Klinicznym MSW, Woloska 137 str., 02-507 W-wa |
| Bioethics Committee at Regional Chamber of Physicians, 5 Gen. J. Zajączka “tr., 42-200 Częstochowa |
| Komisja Bioetyczna przy Uniwersytecie Medycznym w Lublinie, Raclawickie Avenue 1, 20-059 Lublin |
| Komisja Bioetyczna przy Warszawskim Uniwersytecie Medycznym, Żwirki I Wigury 61 str., 02-091 W-wa |
| **SOUTH AFRICA** |
| The South African Medical Association, Block F, Castle Walk Office Park, Nossob Street, Erasmuskloof, Ext 3, Pretoria 0183 SA |
| **UNITED STATES** |
| Sterling IRB, Sally P. Green, MD, 6300 Powers Ferry Road, Suite 600-351, Atlanta, GA 30333 Tel: 770-690-9491  Fax: 770-690-9492 |
| The University of Chicago, Institutional Review Board, Christopher Daugherty, MD, McGiffert Hall, 2nd Floor  5751 S. Woodlawn Avenue, Chicago, IL 60637 |
| Avera IRB, Carol DeSchepper, 3900 West Avera Drive, Sioux Falls, SD 57108 Tel: 605-322-4755  Fax: 605-322-4760 |
| North Memorial Medical Center, 2008 IRB, Irfan Altafullah, MD, 3300 Oakdale Avenue North, Robbinsdale, MN 55422  Tel: 763-520-5200 |
| Western IRB, Theodore D. Schultz, JD, 3535 Seventh Avenue, SW, Olympia, WA 98508 Tel: 800-562-4789  Fax: 360-252-2498 |
| Charleston Area Medical Center W. VA. University IRB, John C. Linton, PhD, 3110 MacCorkle Ave, SE, Rm 3283, Charleston, W.VA. 25304 |
| Genesys Health System IRB, 1 Genesys Parkway, Grand Blanc, MI 48439 |
| Cincinnati Childrens Hospital IRB, 3333 Burnett Ave, Cincinnati, OH 45229 |
| Childrens Mercy Hospital, Pediatric IRB, Office of Research Integrity, 2405 Grand 14th Floor, Kansas City, MO 64108 |
