# Supplementary material for: Cross-reactive immunity potentially drives global oscillation and opposed alternation patterns of seasonal influenza A viruses
Source: Sci Rep. 2022 May 25;12:8883. doi: 10.1038/s41598-022-08233-w (PMC9131982; doi:10.1038/s41598-022-08233-w)
Supplement: Supplementary file 3 — Supplementary Information 2. [file 41598_2022_8233_MOESM3_ESM.docx]

**Fig S1.** Distribution of IRIS centres over the world. IRIS(NCT00884117) is a prospective, multicentre, observational study. This report summarizes the results from 87 centres in, Australia, China (Hong Kong), France, Germany, Poland, Norway, and the United States from December 2008 to March 2013, comprising five Northern and four Southern Hemisphere seasons, and including the 2009–2010 pandemic.

**Fig S2.** Migration network measures and clustering analyses of reconstructed migration networks Complexity of network connections was quantified for A/H3N2/HA (red) and A/pH1N1/HA (blue) using the following measures: (A) Network density. (B) Graph diameter.

(C) Number of islands. (D) Global cluster coefficient.(E) Reciprocity of the graph. Comparison of the connectivity between different geographical locations was done within a 1-year-time-window frame using the following measures: (F) Degree of centrality. (G) Closeness centrality.

(H) Betweenness centrality.

**Fig S3**. Distribution of migration events between geographical locations. Influenza A/H3N2 (A) and A/H1N1 (B) virus migration events were pooled by 1-year intervals. Counts of geographic location switches on the tree were identified and classified using fully-spatiotemporal-resolved HA phylogenies by a discrete source/sink model between centers located in Asia (red), Europe (orange), North America (blue) and Pacific (purple) and are presented in white boxes.

**Fig S4.** Periodograms computed for the reproductive numbers *R-*skylines to estimate oscillation periods and inferred uncertainties. Estimated periods for the A/H1N1 (A) and A/H3N2 (C) reproductive numbers R-skyline plots. Uncertainty quantification on estimated periods for A/H1N1 (B) and A/H3N2 (D) virus was done using cumulative periodograms computed on 100 permutations of the original *R* median time-series.

Supplementary Fig 1


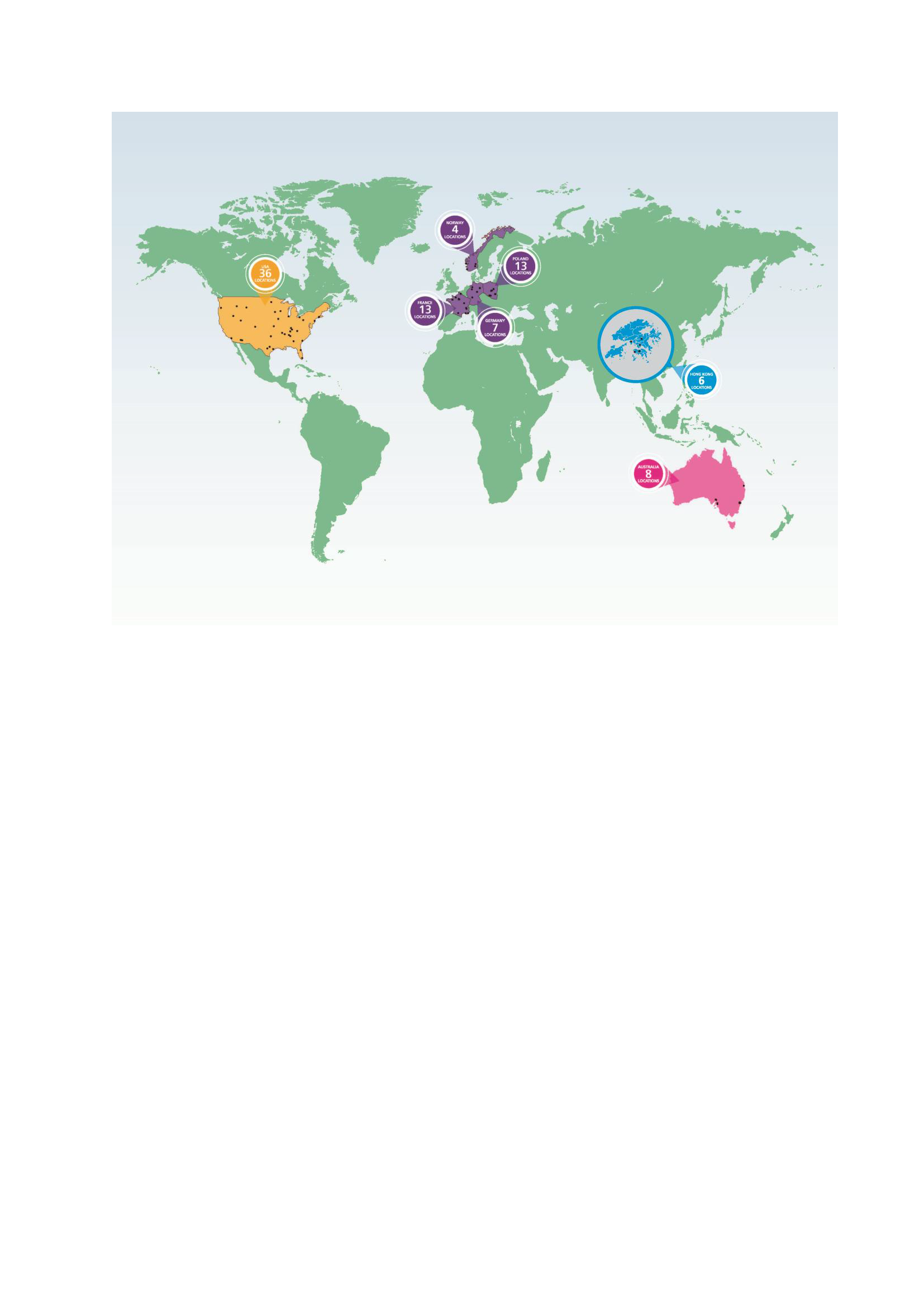


Supplementary Fig 2


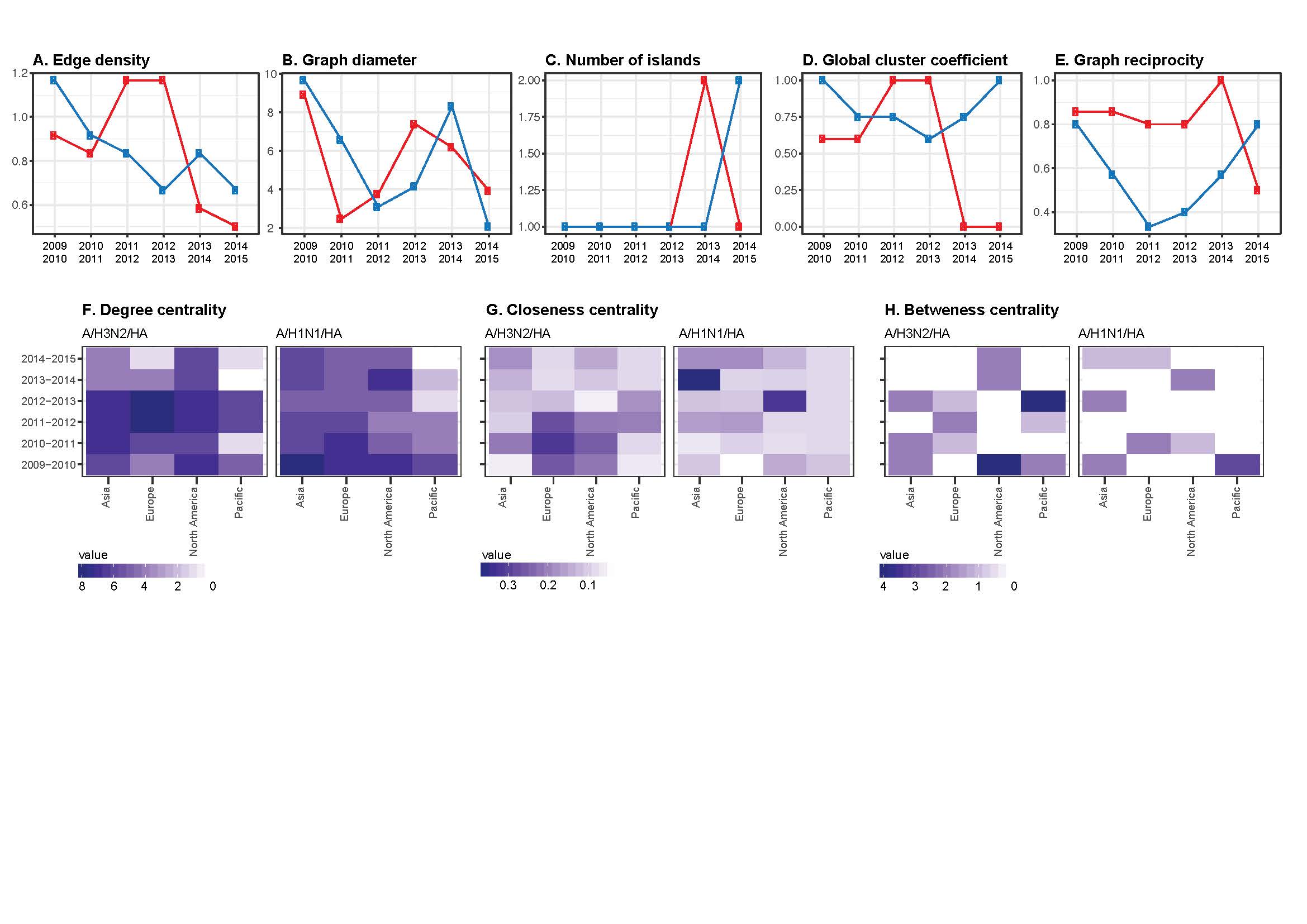


Supplementary Fig 3


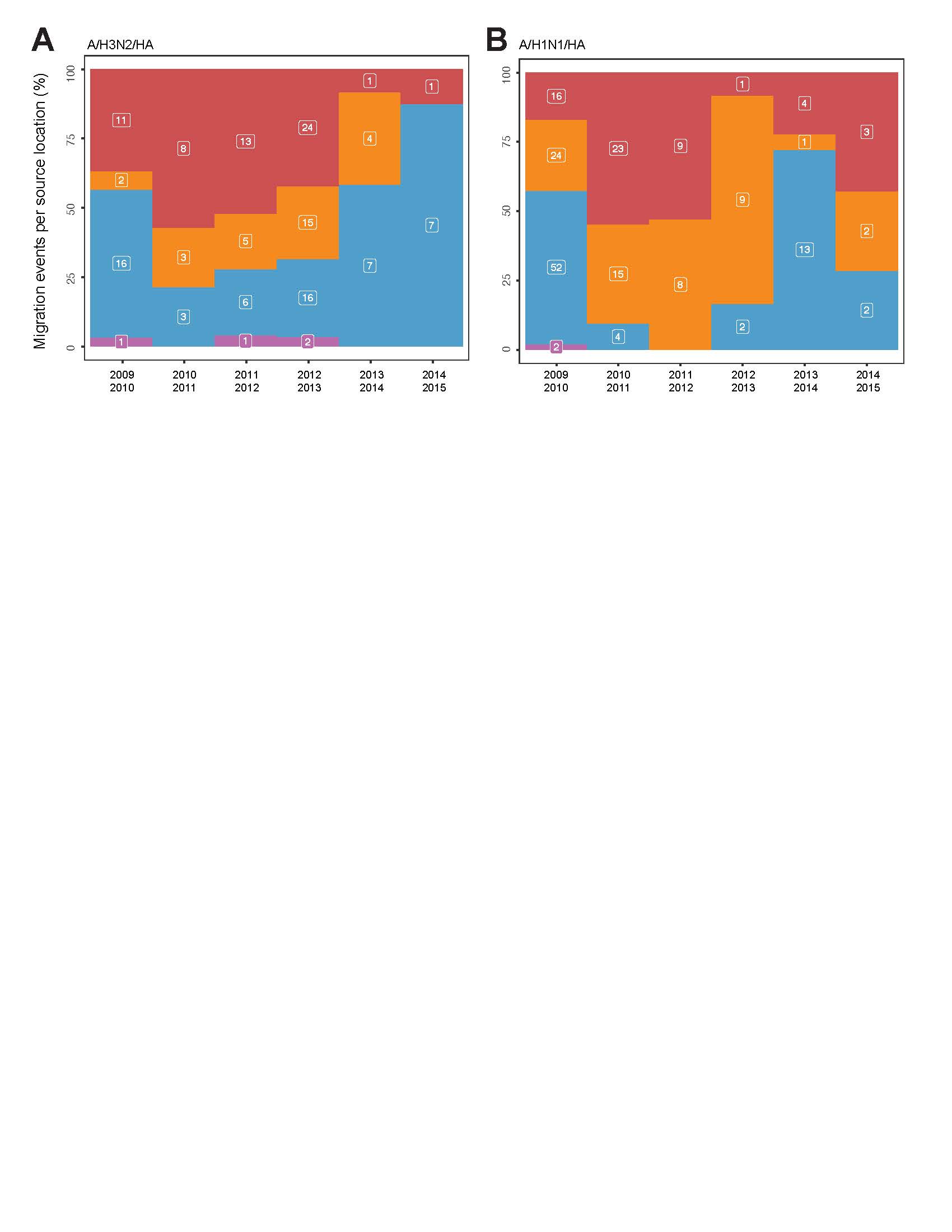


Supplementary Fig 4


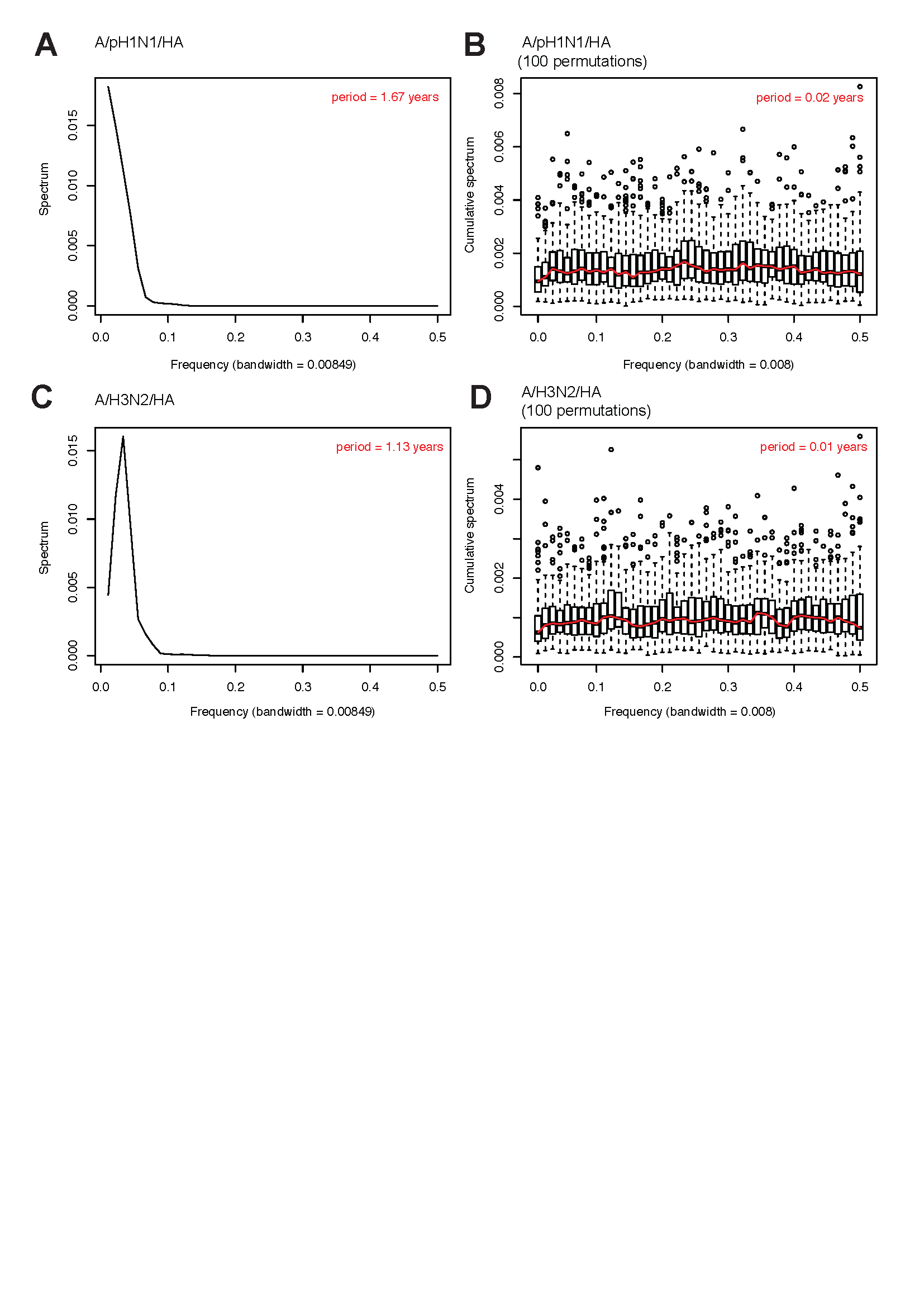


Supplementary material

Data available from the Dryad Digital Repository: http://dx.doi.org/10.5061/dryad.[NNNN]

Supplementary Table 1 Birth-Death Skyline for Influenza A/H1N1 Virus

| Parameter | Mean | Median | SD^e^ | HPD 95%^f^ | ESS^g^ |
| --- | --- | --- | --- | --- | --- |
| R0_1_ ^a^ | 0.9837 | 0.985 | 0.0508 | [0.8793, 1.0824] | 27248 |
| R0_2_ | 0.9849 | 0.9862 | 0.0499 | [0.8823, 1.0824] | 30710 |
| R0_3_ | 0.9846 | 0.9859 | 0.0499 | [0.8842, 1.0842] | 34862 |
| R0_4_ | 0.9851 | 0.9863 | 0.0497 | [0.8841, 1.083] | 27200 |
| R0_5_ | 0.9798 | 0.9814 | 0.0503 | [0.8757, 1.0778] | 25941 |
| R0_6_ | 1.1026 | 1.1012 | 0.0231 | [1.0586, 1.1483] | 542 |
| R0_7_ | 0.9303 | 0.9315 | 0.0167 | [0.8965, 0.9614] | 596 |
| R0_8_ | 1.0000 | 0.9999 | 6.673E-3 | [0.9864, 1.013] | 13635 |
| R0_9_ | 0.9871 | 0.9878 | 0.0162 | [0.9531, 1.0174] | 5158 |
| R0_10_ | 1.0235 | 1.0227 | 0.0108 | [1.003, 1.045] | 1576 |
| δ (days)^b^ | 100.98 (3.61) | 97.47 (3.74) | 22.279 | [2.51, 5.74] | 434 |
| *S* ^c^ | 6.41E-3 | 6.06E-3 | 2.57E-3 | [1.85E-3, 0.01] | 515 |
| Z_0_ (yr)^d^ | 8.331 | 8.330 | 0.017 | [8.298, 8.367] | 2118 |
| Root of the tree (yr) | 3.881 | 3.875 | 0.0421 | [3.812, 3.964] | 1212 |

^a^Effective reproductive number R0_1-10_, ^b^Rate of becoming non-infectious, ^c^Sampling proportion, ^d^Origin of the cluster epidemic, ^e^Standard deviation, ^f^Highest posterior density interval, ^g^Effective sample size.

Supplementary Table 2 Birth-Death Skyline for Influenza A/H3N2 Virus

| Parameter | Mean | Median | SD^e^ | HPD 95%^f^ | ESS^g^ |
| --- | --- | --- | --- | --- | --- |
| R0_1_ ^a^ | 0.977 | 0.9788 | 0.0711 | [0.8312, 1.1167] | 20390 |
| R0_2_ | 1.071 | 1.0677 | 0.0331 | [1.0111, 1.1393] | 1677 |
| R0_3_ | 0.988 | 0.9883 | 0.0221 | [0.9434, 1.0315] | 7145 |
| R0_4_ | 0.936 | 0.9392 | 0.0378 | [0.8595, 1.006] | 1666 |
| R0_5_ | 1.08 | 1.0774 | 0.0241 | [1.0363, 1.1282] | 846 |
| R0_6_ | 0.987 | 0.9874 | 0.0104 | [0.9655, 1.0068] | 3470 |
| R0_7_ | 0.976 | 0.9767 | 0.0168 | [0.9419, 1.0077] | 2349 |
| R0_8_ | 1.057 | 1.0561 | 0.0155 | [1.0277, 1.0873] | 671 |
| R0_9_ | 0.954 | 0.955 | 0.0129 | [0.9279, 0.9773] | 684 |
| R0_10_ | 1.036 | 1.0346 | 0.0113 | [1.0147, 1.058] | 806 |
| δ (days)^b^ | 94.88 (3.85) | 90.72 (4.02) | 23.24 | [2.59, 6.37] | 487 |
| *S* ^c^ | 4.85E-3 | 4.58E-3 | 2.085E-3 | [1.22E-3, 8.99E-3] | 558 |
| Z_0_ (yr)^d^ | 5.084 | 5.085 | 0.0334 | [5.03, 5.1377] | 1307 |
| Root of the tree (yr) | 4.452 | 4.44 | 0.076 | [4.325, 4.603] | 719 |

^a^Effective reproductive number R0_1-10_, ^b^Rate of becoming non-infectious, ^c^Sampling proportion, ^d^Origin of the cluster epidemic, ^e^Standard deviation, ^f^Highest posterior density interval, ^g^Effective sample size.
